# Final outcome of accidents victims referred to emergency departments of hospitals in Iran in 2016

**Published:** 2019-08

**Authors:** Mehrdad Mahdian, Fatemeh Sadat Asgarian, Hossein Akbari

**Affiliations:** ^*a*^Trauma Research Center, Kashan University of Medical Sciences, Kashan, Iran.

## Abstract

**Background:**

Traumas and accidents are the third leading cause of death after cardiovascular disease and cancer. Since the burden of accidents has increased significantly in recent years, therefore, studying the consequences of accidents based on age and sex groups can be effective for health policy making.

**Methods:**

This is a cross-sectional study in which all injured patients referred to the emergency department of hospitals in Iran during 2016 entered the study. The data were collected from the database of injured patients and analyzed by descriptive and analytic statistics using SPSS16 software at error level less than 5%.

**Results:**

In 2016, the 1489862 incidents were recorded, of which the highest number was related to traumatic brain injury (29%), followed by fall (11%). Of the all cases, 5013 (34%) died and 358 cases (0.02%) were reported as disabilities.

**Conclusions:**

In order to reduce the burden of accidents and traumas, comprehensive and interventional programs should be designed and implemented.

**Keywords:**

Accidents, Epidemiology, Injury, Iran, Trauma

